# Supplementary material for: Content-rich biological network constructed by mining PubMed abstracts
Source: BMC Bioinformatics. 2004 Oct 8;5:147. doi: 10.1186/1471-2105-5-147 (PMC528731; doi:10.1186/1471-2105-5-147)
Supplement: Additional File 5 — The original Chilibot query results of the term "long-term potentiation (LTP)" and 22 other terms, limiting the latest references analyzed to the years 1990, 1995, 2000, and 2004. [file 1471-2105-5-147-S5.bz2 › chilibotAdditionalFile5/ltp2004/html/CREB_CAMKII.html]

 


 **CREB** and **CAMKII** 
  
Found 29 abstracts in PubMed,  **29 abstracts were retrieved and analyzed**.  


---

 Search Google  |
 PDF files only 
|  EDU domain only 

---

**Interactive relationship** (e.g. stimulation, inhibition, etc)

- When Ser142 was mutated to alanine,  **CREB**  was activated by  **CaMKII** , as well as by CaMKIV.  Ref: 7958915 Genes Dev, 1994
- We show that i the BDNF stimulated  **CaMKII**  cascade phosphorylates the key positive regulatory site of  **CREB**  via its end MAPKAPK 2 component.  Ref: 12699783 Neuroscience, 2003
- ii this process appears to be highly localized in the outermost cell layer of the dentate gyrus.The present findings suggest that  **CaMKII**  is involved in neurotrophic dependent activation of  **CREB**  in the dentate gyrus.  Ref: 12699783 Neuroscience, 2003
- Thus nuclear  **CaMKII**  modifies neuronal differentiation by a mechanism independent of MAPK and  **CREB**  activation.  Ref: 12570987 Am J Physiol Cell Physiol< MedlineTA>Am J Physiol Cell Physiol, 2003
- However, MEK1 2 inhibitor,  **CaMKII**  inhibitor, and PKC inhibitor significantly suppressed lead induced  **CREB**  phosphorylation.  Ref: 12832008 Brain Res Bull, 2003
- Thus,  **CamKII**  confers a dominant inhibitory effect on transcription by preventing dimerization of  **CREB** , and this mechanism may account for the attenuation of gene expression.  Ref: 11013247 J Biol Chem, 2001
- These results indicate that lead activated PKC,  **CaMKII**  and MEK ERK p90RSK pathways simultaneously, all of which contributed to  **CREB**  phosphorylation.  Ref: 12832008 Brain Res Bull, 2003
- The  **CREB**  CRE transcriptional pathway has been shown to be activated by calcium calmodulin dependent kinase II  **CaMKII**  and mitogen activated protein kinase MAPK.  Ref: 11299324 J Neurochem, 2001
- We conclude that lead activates PKC,  **CaMKII**  or MEK ERK p90RSK pathways in GT1 7 cells, leading to  **CREB**  phosphorylation and modulation of gene expression.  Ref: 12832008 Brain Res Bull, 2003
- **CREB**  in the PSD fraction was phosphorylated both by protein kinase A and calcium calmodulin dependent protein kinase II  **CaMKII**  endogenous to the fraction, and dissociated from the PSD after phosphorylation, especially under  **CaMKII**  activated conditions.  Ref: 9795144 Brain Res Mol Brain Res, 1998
- **CamKII**  modified  **CREB**  binds to DNA efficiently as a monomer.  Ref: 11013247 J Biol Chem, 2001
- ...  **CaMKII**  phosphorylates  **CREB**  at Ser133 and a second site, Ser142.  Ref: 7958915 Genes Dev, 1994
- Here we show that phosphorylation of serine 142 in  **CREB**  by  **CamKII**  leads to dissociation of the  **CREB**  dimer without impeding DNA binding capacity.  Ref: 11013247 J Biol Chem, 2001
- Because both  **CaMKII**  and CaMKIV can phosphorylate  **CREB** , we pursued further the mechanism by which  **CaMKII**  and CaMKIV differentially regulate  **CREB**  activity.  Ref: 7958915 Genes Dev, 1994
- Next, we utilized activators and inhibitors of protein kinase A PKA, protein kinase C PKC, mitogen activated protein kinase kinase MAPKK and calcium calmodulin dependent protein kinase II  [ **CAMKII** ]   **CaMKII**  to determine which kinases might be involved in phosphorylating the  **CREB**  in PZHPV 7 cells.  Ref: 12899525 Recent Results Cancer Res, 2003
- Transient transfection studies revealed that phosphorylation of Ser142 by  **CaMKII**  blocks the activation of  **CREB**  that would otherwise occur when Ser133 is phosphorylated.  Ref: 7958915 Genes Dev, 1994

**Parallel relationship** (e.g. studied together, co-existance, homology, etc.)

- The enhanced learning correlates with increased phosphorylation of cyclic AMP dependent response element binding  **CREB**  protein, of calcium calmodulin dependent protein kinase II  **CaMKII**  and of the GluR1 subunit of the AMPA receptor.  Ref: 12198546 Nature, 2002
- The molecular mechanisms that underlie plastic changes in the barrel cortex still remain unclear, although alpha  **CamKII**  and to lesser extend alpha beta  **CREB**  appear to be involved.  Ref: 9949558 Acta Neurobiol Exp (Wars), 1998
- Group I metabotropic glutamate receptors control phosphorylation of  **CREB** , Elk 1 and ERK via a  **CaMKII**  dependent pathway in rat striatum.  Ref: 11682144 Neurosci Lett, 2001
- These results suggest that calcium calmodulin dependent protein kinase II  [ **CAMKII** ]  induced phosphorylation of  **CREB**  may be involved in regulating feeding behavior induced by NPY.  Ref: 9106624 Mol Pharmacol, 1997
- To evaluate putative roles of calcium calmodulin dependent protein kinase II  **CaMKII**  in  **CREB** , Elk 1 and ERK phosphorylation, the  **CaMKII**  inhibitor, KN62, was infused simultaneously with the group I mGluR agonist, dihydroxyphenylglycine DHPG, into the rat dorsal striatum.  Ref: 11682144 Neurosci Lett, 2001
- These observations show that nociceptive stimuli activate a cholecystokinin pathway from the parabrachial nucleus to the ventromedial hypothalamus that may be important for homeostatic responses to tissue damage, and point to a putative intracellular route for calcium mediated FOS transcription via  **CaMKII**  and  **CREB**  for the regulation of ppCCK transcription.  Ref: 9766403 J Comp Neurol, 1998
- We also investigated V1aR activation of calcium activated signaling kinases, protein kinase C PKC, calcium calmodulin dependent protein kinase II  **CaMKII** , and the mitogen activated protein MAP kinases MAPK and extracellular signal regulated kinases 1 and 2 ERK1 2, their localization within cytoplasmic and nuclear compartments, and activation of their downstream nuclear target, the transcription factor cAMP response element binding protein  **CREB** .  Ref: 12764111 J Neurosci, 2003
- The detection of spatial novelty is associated with a sequential activation of PKA, ERKs p44 and p42 MAPKs and  **CaMKII**  and the phosphorylation of c AMP responsive element binding protein  **CREB**  in the hippocampus.  Ref: 11040265 Learn MemLearn Mem, 2003
- These results suggest that gated induction of Per1 and or Per2 genes through  **CaMKII**   **CREB**  CRE accompanied with photic stimuli may be a critical step in phase shifting.  Ref: 11299324 J Neurochem, 2001
- Additionally, the activations of PKC and p38... calcium calmodulin dependent protein kinase II  [ **CAMKII** ] , and ERK, by C2 ceramide play important regulatory roles in C2 ceramide induced proENK mRNA expression via activating the  **CREB** .  Ref: 11382404 Brain Res, 2001
- The results indicate that CaMKIV is much more potent than  **CaMKII**  in activating  **CREB**  in three different cell lines.  Ref: 7958915 Genes Dev, 1994
- Subsequent to  **CaMKII**  and ERK1 2 translocation to the nucleus,  **CREB**  activation occurred and was found to be dependent on upstream activation of ERK1 2 and CaMKs.  Ref: 12764111 J Neurosci, 2003
- First, that depolarization of SGNs activates CaMKs is evidenced by observation of increased  **CaMKII**  phosphorylation and of CaMK dependent  **CREB**  phosphorylation.  Ref: 12671991 J Neurosci Res, 2003
